# Responsiveness of patient-reported outcomes by disease trajectory in SLE

**DOI:** 10.1093/rheumatology/keag576

**Published:** 2026-10-15

**Authors:** Sabrina Hundal, Julian Cappelli, Mohammed Kaouache, Jessica Muha, Savanah Haidar, Lorenzo Beretta, Zahi Touma, John S Barbieri, Stephanie R Goldberg, Anastasiya Muntyanu, Ioannis Parodis, Elena Netchiporouk

**Affiliations:** Department of Medicine, University of Calgary, Calgary, Alberta, Canada; Division of Experimental Medicine, McGill University Health Centre, Montreal, Quebec, Canada; Department of Health Research Methods, McMaster University, Hamilton, Ontario, Canada; Department of Mathematics and General Sciences, Prince Sultan University, Riyadh, Saudi Arabia; Faculty of Medicine and Health Sciences, McGill University, Montreal, Quebec, Canada; Faculty of Medicine, University of Montreal, Montreal, Quebec, Canada; Referral Center for Systemic Autoimmune Diseases, Fondazione IRCCS Ca’ Granda, Ospedale Maggiore Policlinico di Milano, Milan, Italy; Schroeder Arthritis Institute, Krembil Research Institute, University Health Network, Toronto, Ontario, Canada; Centre for Prognosis Studies in Rheumatic Diseases, University of Toronto Lupus Clinic, Toronto Western Hospital, Toronto, Ontario, Canada; Department of Dermatology, University of Pennsylvania Perelman School of Medicine, Philadelphia, PA, USA; Department of Surgery, Mary Washington Healthcare, Fredericksburg, VA, USA; Division of Dermatology, Department of Medicine, University of Toronto, Toronto, Ontario, Canada; Division of Rheumatology, Department of Medicine Solna, Karolinska Institutet, Karolinska University Hospital, Center for Molecular Medicine (CMM), Stockholm, Sweden; Department of Rheumatology, Faculty of Medicine and Health, Örebro University, Örebro, Sweden; Division of Dermatology, McGill University Health Centre, Montreal, Quebec, Canada

**Keywords:** SLE, patient-reported outcomes, belimumab, responsiveness, outcome measures, quality of life

## Abstract

**Objectives:**

SLE is a heterogeneous autoimmune disease with persistent symptoms that profoundly impair quality of life yet are often under-recognized by clinician-assessed instruments. Patient-reported outcomes (PROs) are increasingly incorporated in clinical trials to capture treatment benefit from the patient perspective. However, the responsiveness of PRO instruments and their alignment with clinician-assessed disease activity across distinct SLE trajectories remain incompletely defined. We evaluated the longitudinal responsiveness and convergent validity of PRO measures across predefined disease trajectory classes.

**Methods:**

Secondary analysis of pooled patient-level data from two phase III randomized clinical trials of belimumab in adults with active SLE (*n* = 1908), stratified into four previously derived trajectory classes defined by baseline disease activity and treatment response. PROs included Functional Assessment of Chronic Illness Therapy–Fatigue (FACIT-Fatigue), 36-Item Short Form Survey (SF-36) Physical Component Summary (PCS) and Mental Component Summary (MCS), EuroQol 5-Dimension 3-level (EQ-5D-3L) and EuroQol Visual Analogue Scale (EQ-VAS). Responsiveness was quantified using standardized response means (SRMs). Convergent validity was evaluated using repeated-measures correlations between within-person changes in PROs and clinician-assessed disease activity measures (SLEDAI-2K, BILAG and Physician’s Global Assessment [PGA]), with sensitivity analyses performed to assess the robustness of findings.

**Results:**

FACIT-F and SF-36 PCS generally demonstrated the greatest responsiveness, while EQ-5D-3L showed smaller changes and less consistent responsiveness across classes and timepoints. FACIT-F demonstrated early responsiveness, whereas SF-36 PCS showed sustained responsiveness over later follow-up. PRO changes were most consistently associated with PGA. Sensitivity analyses showed broadly similar patterns.

**Conclusion:**

FACIT-F and SF-36 PCS demonstrated the greatest responsiveness and alignment with clinician-assessed disease activity. EQ-5D-3L showed less consistent responsiveness, suggesting domain-specific PROs may better capture treatment benefit in SLE trials and health technology assessments.

*Rheumatology* key messages

- FACIT-F and SF-36 PCS demonstrated the strongest responsiveness and clinician-assessed measure alignment in belimumab-treated SLE.
- EQ-5D-3L demonstrated comparatively weaker performance, potentially underestimating clinically meaningful treatment benefit in SLE trials.
- PRO responsiveness varied by disease trajectory and time, supporting trajectory-informed endpoint selection in trials.

## Introduction

SLE is a heterogeneous autoimmune disease that imposes substantial morbidity and excess mortality, with a symptom burden that often persists despite apparent control of clinician-assessed disease activity [1, 2]. Fatigue, pain and limitations in physical and social functioning remain among patients’ most disabling concerns and are frequently under-recognized by commonly used clinician-assessed disease activity instruments, including the SLEDAI 2000 (SLEDAI-2K) and organ-specific instruments [3–5]. Consequently, patient-reported outcomes (PROs) have become integral to trials, clinical care and regulatory and reimbursement decision-making [6].

The interpretability and utility of PROs hinge on responsiveness, defined as the ability of an instrument to detect clinically meaningful within-person change when a change has occurred [7, 8]. Instruments with suboptimal responsiveness may mischaracterize treatment benefit, leading to misleading trial conclusions and potential undervaluation of therapies in economic evaluations [7, 8]. This concern is salient in SLE, where discordance between patient experience and clinician-assessed activity is common [9, 10], and where generic preference-based measures such as EuroQoL-5D (EQ-5D)—central to health technology assessments (HTAs)—may lack sensitivity to change in immune-mediated diseases [11]. Despite widespread use of PROs in SLE, the comparative responsiveness of commonly employed instruments, and whether their performance varies across distinct disease courses, remain incompletely defined.

Belimumab, the first biologic approved for SLE, has consistently reduced disease activity and yielded modest improvements in fatigue and health-related quality of life (HRQoL) across multiple phase III trials [12–15]. Recently, a pooled latent trajectory analysis identified four 52-week disease courses defined by baseline activity and subsequent treatment response [16]. This framework offers a clinically meaningful lens to evaluate PRO responsiveness.

Using patient-level data pooled from two phase III belimumab trials, we sought to: (1) quantify the responsiveness of commonly used PROs over 52 weeks; (2) compare performance across four disease trajectory classes; and (3) examine associations between changes in PROs and changes in clinician-assessed disease activity measures. Our goal was to identify instruments that most reliably capture clinically meaningful change across heterogeneous courses of SLE and to inform endpoint selection for trials, routine care and HTAs.

## Methods

### Study design and data source

We conducted a pooled, patient-level secondary analysis of two multicentre, randomized, double-blind, placebo-controlled phase III trials of belimumab in adults with SLE: BLISS-52 and BLISS-76 (45.30% and 54.70% of participants, respectively) [12, 13]. Full trial protocols, including inclusion and exclusion criteria, randomization procedures, interventions and follow-up schedules, have been published previously [12, 13]. This study was conducted according to the Strengthening the Reporting of Observational Studies in Epidemiology (STROBE) guidelines for observational analyses of randomized clinical trial datasets [17].

### Ethics

Data from the belimumab trials were made available by GlaxoSmithKline (Uxbridge, UK) through the Clinical Study Data Request consortium. The trial protocols were approved by regional ethics review boards for all participating centres and complied with the ethical principles of the Declaration of Helsinki. Written informed consent was obtained from all study participants prior to enrolment. This study was approved by the Swedish Ethical Review Authority (registration number: 2019–05498).

### Participants and eligibility

The parent trials enrolled participants aged ≥18 years who fulfilled the 1997 American College of Rheumatology SLE classification criteria and demonstrated clinically active disease as defined by SLEDAI-2K thresholds and serologic markers [18, 19]. Major exclusions included severe active lupus nephritis and central nervous system lupus. We included all participants randomized to belimumab who had a baseline and at least one post-baseline assessment for at least one eligible PRO through week 52.

### Patient-reported outcomes

PROs were assessed using three validated instruments: the Functional Assessment of Chronic Illness Therapy–Fatigue (FACIT-F); the 36-Item Short Form Survey (SF-36), from which the Physical Component Summary (PCS) and Mental Component Summary (MCS) scores were derived; and the EuroQol 5-Dimension 3-Level (EQ-5D-3L) instrument, including its utility index and accompanying EuroQol Visual Analogue Scale (EQ-VAS). Assessments were conducted at baseline and approximately every 4–12 weeks thereafter (weeks 4, 8, 12, 20, 24, 32, 36, 48 and 52). Higher scores indicate better health status for all PRO measures. To enhance clinical interpretability, mean changes at weeks 24 and 52 were compared with established minimally clinically important differences (MCIDs), including ∼4 points for FACIT-F, 2.5 for SF-36 PCS, 10 for EQ-VAS and 0.04 for EQ-5D-3L [20].

### Clinical instruments

Clinician-assessed disease activity was measured using the SLEDAI-2K, the BILAG index and the Physician’s Global Assessment (PGA). Higher values reflect more active disease. Detailed descriptions of the PRO measures and clinician-assessed disease activity instruments are provided in Table 1.

**Table 1:** Characteristics of patient-reported outcome measures and clinician-assessed disease activity instruments.

Instrument	Domains assessed	Scale	Interpretation of higher scores	Evaluation
Patient-reported outcomes				
FACIT-F	Fatigue and its impact on daily activities	0–52	Less fatigue	Validated in SLE; sensitive to short-term fatigue changes
SF-36 PCS	Physical functioning, role limitations due to physical health, pain, general health	0–100 (normed to mean 50, SD 10)	Better physical health and function	Captures physical function and pain; responsive to treatment over longer intervals
SF-36 MCS	Vitality, social functioning, role limitations due to emotional problems, mental health	0–100 (normed to mean 50, SD 10)	Better mental health and function	Psychological and emotional health; less consistently responsive in SLE trials
EQ-5D-3L	Mobility, self-care, usual activities, pain/discomfort, anxiety, depression	0 = death; 1 = perfect health; <0 = worse than death	Better health state	Utility index; QALY estimation in economic evaluation; limited sensitivity in immune diseases
EQ-VAS	Self-rated overall health status	0–100	Better perceived overall health	Concise, global measure of health; variable correlations with disease activity
Clinician-assessed measures				
SLEDAI-2K	Clinical and laboratory manifestations across multiple organ systems	0–105	More active disease	Heavily weighted towards serological activity; widely used trial endpoint
BILAG	Organ-specific disease activity	Categories (A–E), converted numerical	More active disease	Comprehensive multi-organ involvement; detailed but resource-intensive
PGA	Overall physician-rated disease activity	0–3 visual analogue scale	More active disease	Simple global measure; consistently aligns with PROs

Domains assessed, scoring ranges, interpretation of higher scores and evaluation notes are summarized for each patient-reported outcome and clinician-assessed index included in this analysis. Instruments are described with respect to their measurement focus, scale properties and relevance in SLE trials. EQ-5D-3L: EuroQol 5-Dimension 3-Level; EQ-VAS: EuroQol Visual Analogue Scale; FACIT-F: Functional Assessment of Chronic Illness Therapy–Fatigue; MCS: Mental Component Summary; PCS: Physical Component Summary; PGA: Physician’s Global Assessment; PRO: patient-reported outcome; QALY: quality-adjusted life-year; SD: standard deviation; SF-36: 36-Item Short Form Survey; SLEDAI-2K: SLEDAI 2000.

### Disease trajectory classification

To account for heterogeneity in disease course after treatment initiation, we applied previously derived disease-trajectory classes generated using growth mixture modelling of the pooled belimumab trial data [16]. These latent trajectories were defined along two dimensions: baseline disease activity (high vs moderate) and subsequent treatment response (responder vs non-responder), yielding four classes: highly active non-responders (class 1), highly active responders (class 2), moderately active responders (class 3) and moderately active non-responders (class 4) [16].

Class assignment was primarily informed by longitudinal patterns in clinician-assessed disease activity measures, including SLEDAI-2K, BILAG and PGA. FACIT-F was included in the original trajectory model but contributed substantially less to trajectory classification than clinician-assessed measures, accounting for 5.79% to 12.80% of the explained variance in trajectory membership across timepoints [16]. For the base case analysis, we used the previously established trajectory assignments without re-fitting the mixture model. To assess potential circularity from inclusion of FACIT-F, we performed sensitivity analyses using trajectory classes redefined after excluding FACIT-F.

### Outcomes

The primary outcome was longitudinal responsiveness of each PRO over 52 weeks, quantified using the standardized response mean (SRM), defined as the mean within-person change from baseline divided by the standard deviation (SD) of that change [21]. SRMs provide a distribution-based measure of sensitivity to change that facilitates comparison across instruments with different scaling properties [22]. SRMs were calculated for the pooled cohort and separately within each class at each trial time point. The secondary outcome was concurrent convergent validity, evaluated by examining the association between within-person changes in PRO scores and simultaneous changes in clinician-assessed disease activity measures over follow-up.

### Statistical analysis

SRMs were calculated as the mean within-person change from baseline divided by the SD of the corresponding difference scores. SRMs and corresponding 95% confidence intervals (CIs) were estimated using the cohens_d function in the R package effectsize, with paired = TRUE; CIs were estimated using the package-default noncentral t-distribution method. Effect size magnitudes were interpreted using conventional thresholds (0.2, small; 0.5, moderate; 0.8, large) [21]. PRO responsiveness was summarized in both early (≤24 weeks) and later (24–52 weeks) intervals to align with known treatment-response dynamics in belimumab trials [23].

To evaluate convergent validity, repeated-measures correlation analyses were used to assess within-subject associations between changes in PROs and concurrent changes in clinician-assessed disease activity measures across repeated observations. Negative correlation coefficients indicate that increases in disease activity were associated with worsening PRO scores.

Where hypothesis testing was performed, tests were two-sided with a significance level of α = 0.05. Analyses were conducted using R version 4.5.1 (R Foundation for Statistical Computing), including repeated-measures correlation modelling (rmcorr).

### Missing data handling

Missing data were addressed using visit-specific complete-case analysis, consistent with recommended practice for change-score–based responsiveness analyses [7, 24]. For each post-baseline timepoint, participants were included if both baseline and the corresponding post-baseline PRO assessment were available. Baseline characteristics were compared between participants with complete vs incomplete 52-week follow-up using Wilcoxon rank-sum tests for continuous variables and chi-squared tests for categorical variables.

### Sensitivity analyses

Sensitivity analyses were performed to assess the robustness of findings to missing data and trajectory-class derivation. First, multivariate imputation by chained equations (MICE) was performed using the *mice* package in R. Five imputed datasets were generated separately within each trajectory class. Predictive mean matching was used for continuous variables. The imputation model included age, sex and baseline and follow-up scores; no additional auxiliary variables were included. SRMs and repeated-measures correlations were re-estimated in each imputed dataset using the same methods as in the primary analysis. Within-imputation variances were derived from the corresponding 95% CIs, with the standard error approximated as the interval width divided by 2 × 1.96. Within- and between-imputation variances were then combined using Rubin’s rules to estimate total variance and 95% CIs.

Second, to minimize potential circularity between trajectory derivation and outcome assessment, disease trajectory classes were redefined using a latent class model based exclusively on clinician-assessed disease activity measures, with adjustment for Systemic Lupus International Collaborating Clinics Damage Index (SLICC-DI) and treatment arm. PROs, including FACIT-F, were excluded from this class derivation.

## Results

### Study population

A total of 1908 participants with active, autoantibody-positive SLE contributed evaluable PRO and disease activity data. Trajectory classification identified 274 participants (14.36%) in class 1 (highly active non-responder), 259 (13.57%) in class 2 (highly active responder), 254 (13.31%) in class 3 (moderately active responder) and 1121 (58.75%) in class 4 (moderately active non-responder). Baseline demographic and clinical features differed across classes, with class 1 exhibiting the highest disease activity (mean SLEDAI-2K 12.76 [SD 4.05]) and the lowest FACIT-F (25.52 [11.96]) and SF-36 PCS (34.77 [9.26]) scores, reflecting worse HRQoL (Table 2). No patients met criteria for Lupus Low Disease Activity State or remission at baseline.

**Table 2:** Baseline demographic and clinical characteristics by disease trajectory class.

Variable	Mean [SD]				
	Class 1	Class 2	Class 3	Class 4	All classes
Disease activity	Highly active	Highly active	Moderately active	Moderately active	Pooled data
Response	Non-responder	Responder	Responder	Non-responder	
Sample (%)	274 (14.36%)	259 (13.57%)	254 (13.31%)	1121 (58.75%)	1908
Females (%)	263 (95.99%)	241 (93.05%)	241 (94.88%)	1064 (94.92%)	1809 (94.8%)
Males (%)	11 (4.01%)	18 (6.95%)	13 (5.12%)	57 (5.08%)	99 (5.19%)
Age [SD]	37.74 [11.28]	36.89 [11.66]	38.50 [11.75]	38.24 [11.46]	38.02 [11.50]
Disease duration (years)	6.93 [6.23]	6.29 [6.67]	5.91 [6.25]	6.55 [6.63]	6.48 [6.53]
Prednisone (mg/day)	10.88 [8.74]	11.42 [8.93]	10.75 [8.73]	10.99 [8.99]	11.00 [8.90]
BILAG	22.47 [6.97]	20.65 [8.53]	16.04 [7.60]	14.79 [7.02]	16.85 [7.91]
SLEDAI-2K	12.76 [4.05]	12.32 [4.12]	9.96 [3.04]	9.22 [3.06]	10.25 [3.67]
PGA	1.75 [0.44]	1.63 [0.46]	1.47 [0.48]	1.37 [0.45]	1.48 [0.48]
FACIT-F	25.52 [11.96]	30.31 [11.78]	31.33 [12.33]	31.50 [11.71]	30.45 [12.01]
SF-36 PCS	34.77 [9.26]	39.23 [9.36]	40.50 [9.24]	39.92 [9.54]	39.25 [9.60]
SF-36 MCS	37.35 [10.71]	38.55 [10.35]	40.96 [10.08]	41.87 [11.25]	40.84 [11.07]
EQ-5D-3L	0.67 [0.21]	0.75 [0.15]	0.73 [0.18]	0.76 [0.18]	0.74 [0.18]
EQ-VAS	56.25 [19.65]	61.81 [19.37]	64.67 [21.35]	64.88 [19.41]	63.42 [19.88]

Baseline demographic variables and mean scores for clinician-assessed instruments and patient-reported outcomes are presented for each of the four latent disease trajectory classes and for the pooled population. EQ-5D-3L: EuroQol 5-Dimension 3-Level; EQ-VAS: EuroQol Visual Analogue Scale; FACIT-F: Functional Assessment of Chronic Illness Therapy–Fatigue; HRQoL: health-related quality of life; MCS: Mental Component Summary; PCS: Physical Component Summary; PGA: Physician’s Global Assessment; PRO: patient-reported outcome; QALY: quality-adjusted life years; SD: standard deviation; SF-36: 36-Item Short Form Survey; SLEDAI-2K: SLEDAI 2000.

### Responsiveness

Across the pooled cohort, FACIT-F and SF-36 PCS demonstrated the largest SRMs over 52 weeks, whereas EQ-5D-3L showed smaller changes relative to other PROs (Fig. 1). Responsiveness varied substantially across the four disease trajectory classes.

**Figure 1:**
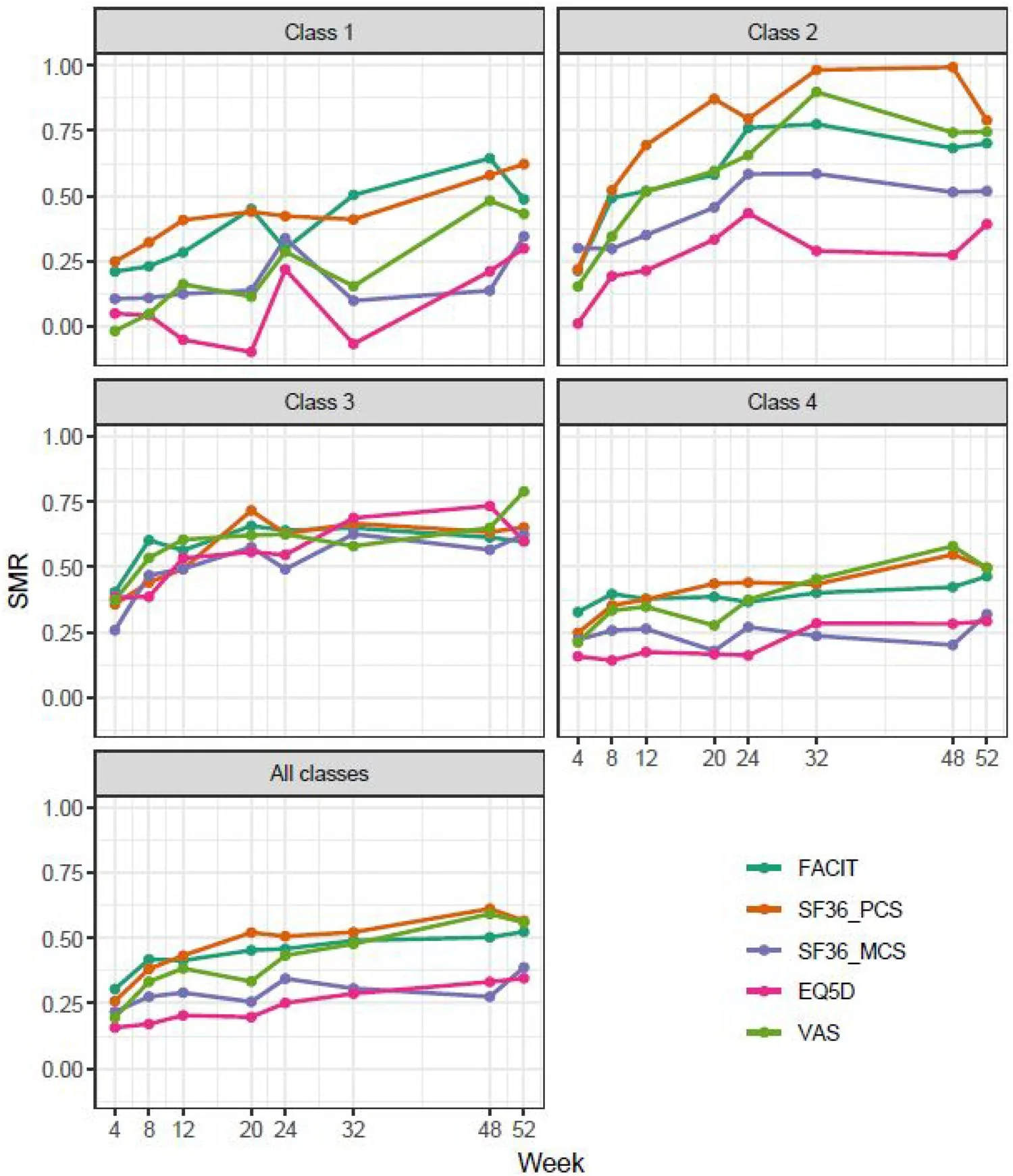
Standardized response means for patient-reported outcomes over 52 weeks, overall and by disease trajectory class. Standardized response means (SRMs) for patient-reported outcomes at study visits from week 4 through week 52, stratified by disease trajectory class (classes 1–4) and for the overall cohort (‘All Classes’). SRMs are defined as the mean within-person change from baseline divided by the standard deviation of that change. Higher absolute SRM values indicate greater responsiveness. Plots display longitudinal responsiveness for the FACIT-F, SF-36 PCS, SF-36 MCS, EQ-5D-3L and EQ-VAS. Only participants with paired baseline and post-baseline observations contributed to visit-specific estimates. EQ-5D: EuroQol 5-Dimension; FACIT-F: Functional Assessment of Chronic Illness Therapy–Fatigue; MCS: Mental Component Summary; PCS: Physical Component Summary; PGA: Physician’s Global Assessment; PRO: patient-reported outcome; SF-36: 36-Item Short Form Survey; SRM: standardized response mean; VAS: Visual Analogue Scale

Among highly active non-responders (class 1), FACIT-F showed the largest SRM at week 48 (0.64), followed by SF-36 PCS at week 52 (0.62), while EQ-5D-3L did not exceed 0.30 at any timepoint. In highly active responders (class 2), SF-36 PCS exhibited the greatest responsiveness, peaking at week 48 (SRM 0.99) and FACIT-F reached 0.68 at the same timepoint. In moderately active responders (class 3), EQ-VAS demonstrated the largest SRM at week 52 (0.79), followed by SF-36 PCS (0.65), while FACIT-F peaked earlier at week 20 (0.66). Among moderately active non-responders (class 4), SF-36 PCS and EQ-VAS showed the highest responsiveness at week 48 (0.55 and 0.58, respectively). Overall, FACIT-F demonstrated early responsiveness, particularly among moderately active responders, whereas SF-36 PCS showed strong and sustained responsiveness over later follow-up, most notably in the highly active trajectory classes. In moderately active responders, later responsiveness was observed across multiple PRO instruments (Fig. 2). Visit-specific SRMs for all instruments and trajectory classes are provided in Table S1.

**Figure 2:**
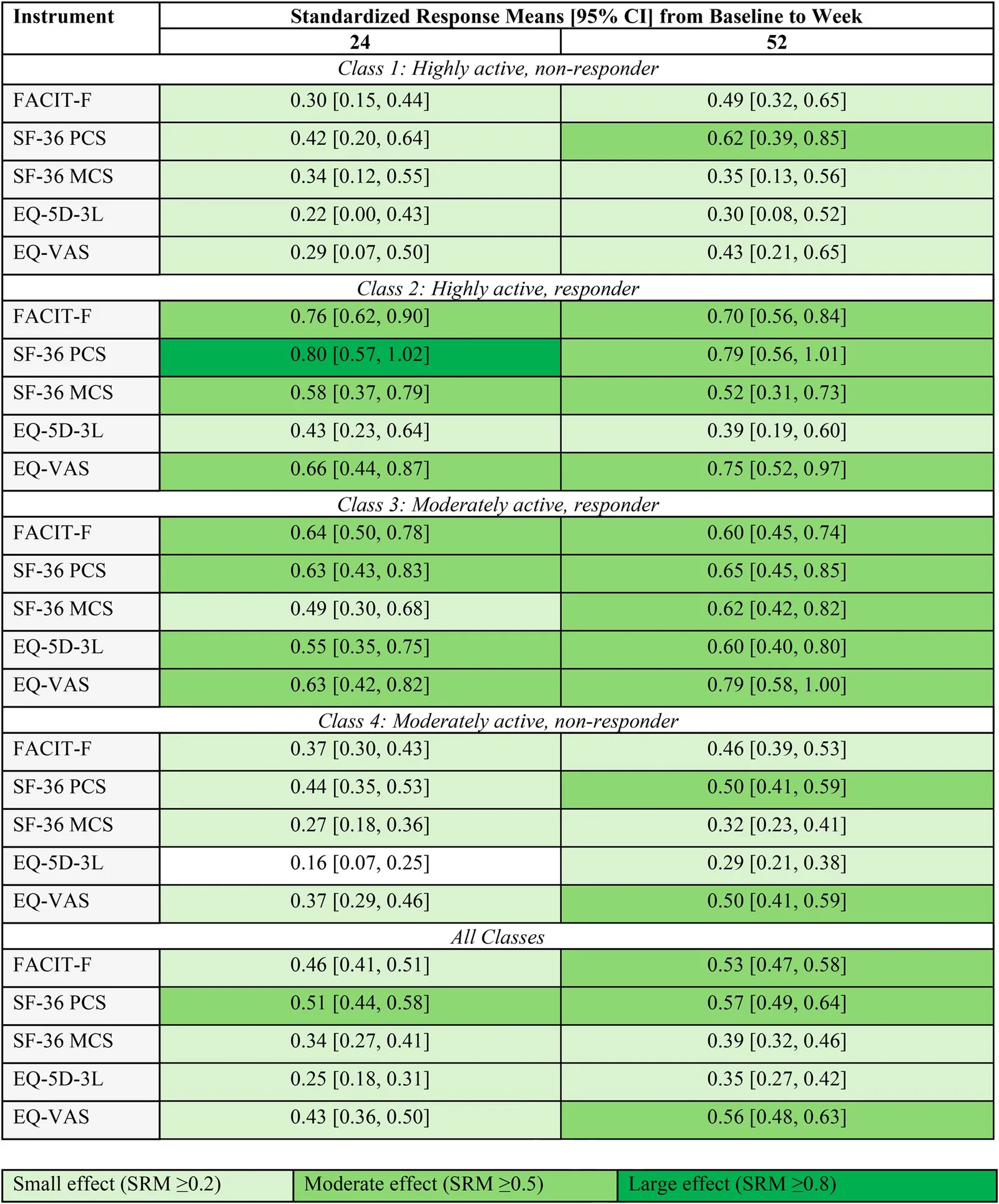
Responsiveness of patient-reported outcomes at weeks 24 and 52, stratified by disease trajectory class. Standardized response means for patient-reported outcomes at weeks 24 and 52 in the overall cohort and stratified by disease trajectory class (class 1, highly active non-responder; class 2, highly active responder; class 3, moderately active responder; class 4, moderately active non-responder). Effect size magnitude is indicated by colour coding: 0.2 or greater, small; 0.5 or greater, moderate; and 0.8 or greater, large. CI: confidence interval; EQ-5D-3L: EuroQol 5-Dimension 3-Level; EQ-VAS: EuroQol Visual Analogue Scale; FACIT-F: Functional Assessment of Chronic Illness Therapy–Fatigue; MCS: Mental Component Summary; PCS: Physical Component Summary; PGA: Physician’s Global Assessment; PRO: patient-reported outcome; SF-36: 36-Item Short Form Survey; SRM: standardized response mean

### Correlation

Repeated-measures correlations between changes in PROs and clinician-assessed disease activity measures are presented in Fig. 3. Across trajectory classes, changes in PROs were most strongly associated with changes in PGA, with weaker and more variable associations observed for SLEDAI-2K and BILAG.

**Figure 3:**
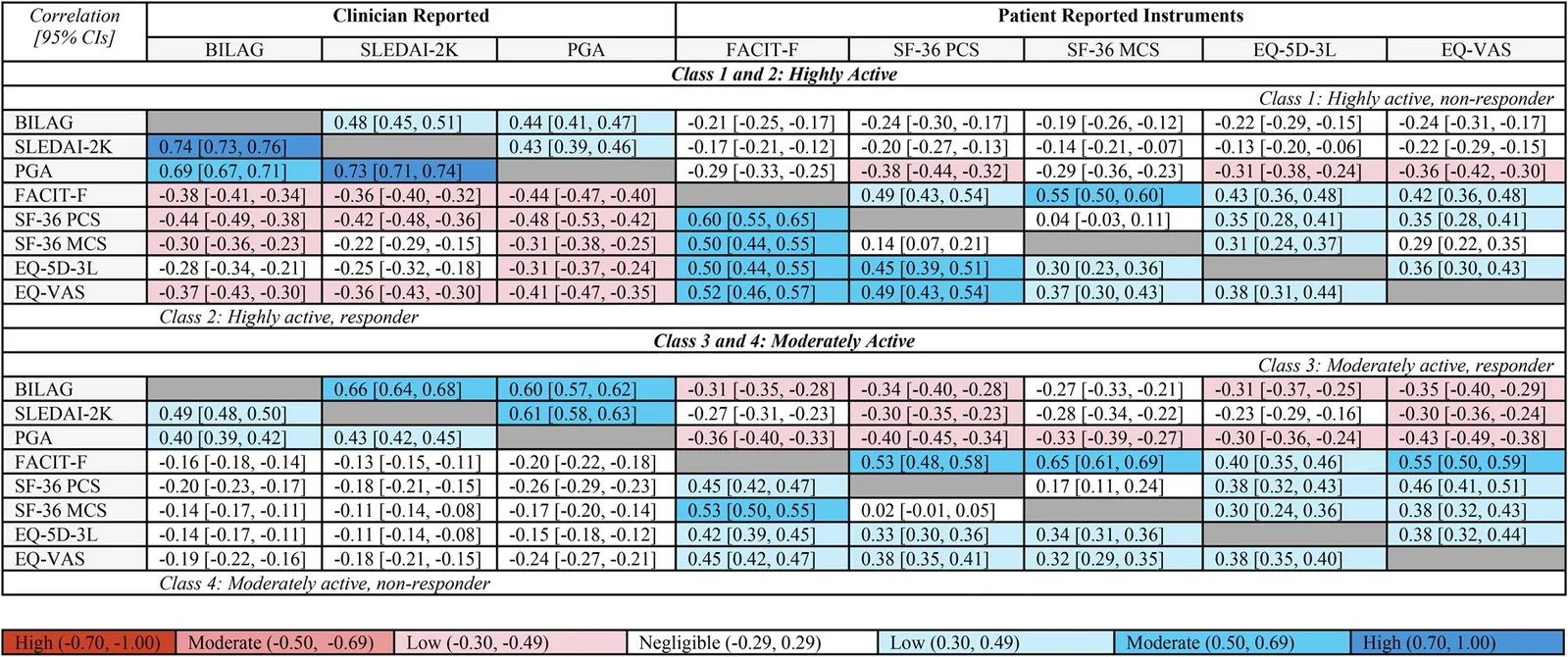
Repeated-measures correlations between changes in patient-reported outcomes and clinician-assessed disease activity measures, by disease trajectory class. Values represent repeated-measures correlation coefficients [95% confidence intervals] between changes in patient-reported outcomes and concurrent changes in clinician-assessed disease activity measures from baseline through week 52. Results are stratified by disease trajectory class. For highly active groups (classes 1 and 2), class 1 (highly active non-responder) values are shown in the upper triangle and class 2 (highly active responder) values in the lower triangle. For moderately active groups (classes 3 and 4), class 3 (moderately active responder) values are presented in the upper triangle and class 4 (moderately active non-responder) values in the lower triangle. Negative correlation coefficients indicate that increases in disease activity are associated with worsening patient-reported outcomes. EQ-5D-3L: EuroQol 5-Dimension 3-Level; EQ-VAS: EuroQol Visual Analogue Scale; FACIT-F: Functional Assessment of Chronic Illness Therapy–Fatigue; MCS: Mental Component Summary; PCS: Physical Component Summary; PGA: Physician’s Global Assessment; PRO: patient-reported outcome; SF-36: 36-Item Short Form Survey; SLEDAI-2K: SLEDAI 2000

In highly active responders (class 2), correlations between PGA and PRO change scores ranged from −0.31 for EQ-5D-3L and SF-36 MCS to −0.48 for SF-36 PCS; FACIT-F demonstrated a correlation of −0.44 with PGA. In moderately active responders (class 3), PGA correlations ranged from −0.30 for EQ-5D-3L to −0.43 for EQ-VAS.

In non-responder classes, correlations were generally smaller in magnitude. In highly active non-responders (class 1), correlations between PGA and PRO changes ranged from −0.29 for FACIT-F and SF-36 MCS to −0.38 for SF-36 PCS. In moderately active non-responders (class 4), correlations between PRO changes and clinician-assessed measures did not exceed −0.26 in magnitude. Across trajectory groups, EQ-5D-3L demonstrated smaller correlations with clinician-assessed disease activity compared with FACIT-F and SF-36 PCS.

### Clinical interpretability using MCID thresholds

Mean changes from baseline in PRO scores at weeks 24 and 52 were interpreted relative to established MCID thresholds (Table S2). In responder trajectories, FACIT-F and SF-36 PCS met or exceeded MCIDs at both timepoints: FACIT-F improved by 6.00–7.08 points, exceeding the 4-point MCID, and SF-36 PCS improved by 4.00–7.00 points, exceeding the 2.5-point MCID. In non-responder trajectories, improvements were smaller but still clinically meaningful for SF-36 PCS at both timepoints and for FACIT-F in class 1 at both timepoints and class 4 at week 52. EQ-VAS met the 10-point MCID in class 3 at both timepoints and class 2 at week 52, while EQ-5D-3L met the 0.04 MCID at most timepoints except class 2 at week 52 and class 4 at week 24.

### Missing data

Data completeness declined from 100% at baseline to 78.98% at week 52 (Table S3). Participants with incomplete 52-week follow-up differed from those with complete follow-up on several baseline characteristics, including BILAG (*P* = 0.03), SLEDAI-2K (*P* = 0.02), FACIT-F (*P* = 0.001), SF-36 PCS (*P* < 0.001), EQ-5D-3L (*P* < 0.001), EQ-VAS (*P* = 0.05) and age (*P* = 0.03) (Table S4).

### Sensitivity analyses

Sensitivity analyses demonstrated findings broadly consistent with the base case analyses. In MICE analyses, SRMs were similar across instruments and trajectory classes, with no meaningful differences in effect size magnitude or temporal patterns (Table S5). Repeated-measures correlations between PROs and clinician-assessed disease activity measures were generally attenuated in magnitude, although the directionality of associations remained unchanged (Table S6).

When disease trajectory classes were redefined after excluding FACIT-F from class derivation, overall patterns of responsiveness were preserved. SRMs were higher across several instruments in the highly active non-responder group (class 1), including FACIT-F at week 52 (0.71 vs 0.49 in the base case) and SF-36 PCS at week 52 (0.88 vs 0.62) (Fig. S1 and Table S5). Repeated-measures correlations showed broadly similar direction and interpretation, with PGA remaining among the clinician-assessed measures most consistently associated with PRO changes across trajectory groups (Table S7).

## Discussion

In this pooled patient-level analysis of two phase III belimumab trials, commonly used PRO instruments differed meaningfully in their responsiveness to clinical change in SLE, and these differences varied by disease trajectory and timing of follow-up. In an analytic cohort of 1908 participants assessed using visit-specific follow-up through week 52, FACIT-F and SF-36 PCS generally demonstrated greater sensitivity to within-person change, whereas EQ-5D-3L showed smaller changes relative to other PRO instruments. These findings highlight the importance of selecting domain-specific PROs capable of capturing patient-valued improvements in fatigue and physical functioning, and they underscore the limitations of generic preference-based instruments when used as primary indicators of treatment benefit in SLE.

Prior work has documented discordance between clinician-assessed disease activity and patient experiences of fatigue, physical impairment and mental well-being [9, 10]. However, most prior studies evaluated PROs cross-sectionally or across mixed patient populations, obscuring heterogeneity in treatment response. By stratifying outcomes according to previously defined disease trajectory classes, our analysis demonstrates that PRO performance is sensitive to baseline disease severity, subsequent response to therapy and phase of follow-up. FACIT-F demonstrated early responsiveness, whereas SF-36 PCS showed strong and sustained responsiveness over later follow-up, most notably in highly active trajectory classes. These findings suggest that fatigue, physical function, mental health and global health status capture related but distinct dimensions of patient-relevant treatment benefit and that no single PRO fully captures the spectrum of health changes experienced by patients with SLE.

Our findings parallel evidence from other immune-mediated inflammatory diseases, where SF-36 PCS and FACIT-F outperform EQ-5D-3L in capturing clinically meaningful changes [11, 25]. In SLE, recent analyses have shown that fatigue frequently improves despite limited changes in serologic or laboratory-weighted measures [26]. This reinforces the need for PRO selections that reflect symptom domains most relevant to patients. Incorporating both FACIT-F and SF-36 PCS could better identify residual disease burden that is not adequately captured by SLEDAI-2K or BILAG, supporting more patient-centred assessment of treatment response. Given that SLE is a complex multisystem disease with cumulative and often irreversible consequences, HRQoL impairment may persist even when inflammatory activity is controlled. This complexity likely contributes to the weaker and less consistent responsiveness of generic utility measures in SLE.

FACIT-F and SF-36 PCS not only demonstrated larger SRMs but also met or exceeded MCID thresholds in most class-by-timepoint comparisons, particularly among responder trajectories, suggesting that observed changes were clinically meaningful. In contrast, EQ-VAS and EQ-5D-3L showed less consistent MCID attainment across classes and timepoints. These findings support FACIT-F and SF-36 PCS as the most responsive measures among those evaluated, with changes that were most consistently aligned with established MCID thresholds, particularly in responder trajectories.

Given FACIT-F contributed to the original trajectory classification, we performed a sensitivity analysis in which trajectory classes were redefined after excluding FACIT-F from class derivation. Overall patterns of responsiveness were preserved, although SRMs were higher across several instruments in the highly active non-responder group. Repeated-measures correlations showed broadly similar direction and interpretation, suggesting that the main findings were not driven by potential circularity from inclusion of FACIT-F in the original trajectory model. Similarly, multiple imputation analyses showed SRMs comparable to the base case analyses. Repeated-measures correlations were generally attenuated after imputation, but the direction of associations remained unchanged. Collectively, these analyses strengthen confidence that the observed patterns were not substantially explained by missing data or by inclusion of FACIT-F in the original class derivation.

EQ-5D-3L generally showed smaller changes than other PRO instruments and weaker correlations with clinician-assessed disease activity. This has important implications for reimbursement decisions, as EQ-5D utilities are central to HTAs. Although EQ-5D-3L met MCID thresholds in some trajectory classes and timepoints, its responsiveness was less consistent than that of FACIT-F and SF-36 PCS, suggesting that reliance on EQ-5D-3L alone may underestimate patient-valued treatment benefits in SLE. Recent work has suggested that categorical EQ-5D ‘full health state’ classifications may discriminate between responders and non-responders [27, 28]. However, our findings suggest that continuous EQ-5D-3L utility scores may be less sensitive to within-person treatment-related change. Algorithms mapping domain-specific PROs to utility measures may offer a pragmatic solution but require validation in diverse SLE cohorts.

PGA demonstrated the strongest and most consistent alignment with PRO changes, whereas SLEDAI-2K and BILAG demonstrated weaker correlations, particularly among highly active non-responders. This suggests that global clinician assessment and patient-reported burden may capture overlapping dimensions of disease impact not fully reflected by laboratory-weighted or organ-specific measures [29, 30]. These findings support composite endpoints that integrate PROs with clinician-assessed measures to better reflect therapeutic impact in SLE [31].

### Strengths and limitations

This study has several strengths. First, the analysis used patient-level data pooled from two pivotal, similarly designed randomized trials, allowing harmonized assessment of PRO responsiveness across a large analytic cohort with standardized follow-up and outcome measurement. Second, the use of predefined trajectory classes enabled evaluation of PRO performance across clinically meaningful patterns of baseline activity and treatment response, rather than assuming uniform responsiveness across all SLE patients. Third, repeated-measures correlation analyses allowed assessment of within-subject associations between changes in PROs and clinician-assessed disease activity across repeated observations. Finally, the robustness of the findings was supported by sensitivity analyses using multiple imputation for missing data and alternative trajectory classes derived after excluding FACIT-F.

Several limitations should also be considered. First, these analyses were *post hoc* and based on participants enrolled in clinical trials, which may limit generalizability to real-world SLE populations with greater comorbidity burden, treatment heterogeneity and variable adherence or persistence. Second, the base case analyses used visit-specific complete-case methods. Although multiple imputation yielded broadly consistent findings, participants with incomplete 52-week follow-up differed from those with complete follow-up on several baseline characteristics, and residual bias from non-random missingness cannot be excluded. Third, trajectory classification was derived from prior modelling of trial data, and although sensitivity analyses excluding FACIT-F from class derivation supported the overall findings, some potential dependence between trajectory assignment and PRO responsiveness remains possible. Fourth, the PRO instruments were limited to those collected in the BLISS trials, and lupus-specific measures such as LupusQoL or LupusPRO, as well as EQ-5D-5L, could not be evaluated. Finally, follow-up was limited to 52 weeks, and longer-term patterns of PRO responsiveness, particularly in relation to sustained disease control, damage accrual and treatment persistence, remain uncertain.

## Conclusion

In this pooled patient-level analysis of two phase III belimumab trials, PRO responsiveness varied by disease trajectory and timing of follow-up. FACIT-F and SF-36 PCS demonstrated the most consistent responsiveness among the measures evaluated, with changes that aligned most closely with established MCID thresholds, particularly in responder trajectories. In contrast, EQ-5D-3L showed smaller changes in the pooled cohort and less consistent responsiveness across trajectory classes and timepoints, despite meeting MCID thresholds in some comparisons.

These findings have implications for both SLE trial design and HTA. Because EQ-5D utilities are commonly used to estimate quality-adjusted life-years and inform reimbursement decisions, reliance on EQ-5D-3L alone may underrepresent patient-valued treatment benefits, particularly improvements in fatigue and physical functioning. Domain-specific measures such as FACIT-F and SF-36 PCS may therefore provide important complementary information when evaluating treatment benefit in SLE.

Future work should validate approaches for translating domain-specific PRO improvements into utility estimates, evaluate PRO responsiveness in real-world and longer-term SLE cohorts and determine whether composite endpoints integrating patient-reported measures with clinician-assessed disease activity better capture meaningful treatment benefit.

## Supplementary Material

keag576_Supplementary_Data

## Data Availability

The datasets used and analysed during this study are available from the corresponding author upon reasonable request.
