# Supplementary material for: Responsiveness of patient-reported outcomes by disease trajectory in SLE
Source: Rheumatology (Oxford). 2026 Oct 15;65(10):keag576. doi: 10.1093/rheumatology/keag576 (PMC13693596; doi:10.1093/rheumatology/keag576)
Supplement: Supplementary file 1 [file keag576_supplementary_data.docx]

**Supplementary Appendix**

**Supplementary Table S1.** Standardized Response Means for Patient-Reported Outcomes by Study Visit, Stratified by Disease Trajectory Class

**Supplemental Table S2.** Mean Changes in Patient-Reported Outcomes Relative to Established Minimal Clinically Important Difference Thresholds

**Supplementary Table S3.** Participant Follow-up and Data Availability by Study Visit

**Supplementary Table S4.** Baseline Participant Characteristics for Complete versus Incomplete Follow-up

**Supplementary Table S5.** Responsiveness of Patient-Reported Outcomes at Weeks 24 and 52 Using Base Case, Multiple Imputation, and Classes Excluding FACIT-F from Trajectory Derivation

**Supplementary Table S6.** Repeated-Measures Correlations Between Patient-Reported Outcomes and Disease Activity: Sensitivity Analysis using Multiple Imputation

**Supplementary Table S7.** Repeated-Measures Correlations Between Patient-Reported Outcomes and Disease Activity: Sensitivity Analysis Excluding FACIT-F from Trajectory Class Derivation

**Supplementary Figure S1.** Standardized Response Means for Patient-Reported Outcomes Over 52 Weeks: Sensitivity Analysis Excluding FACIT-F from Trajectory Class Derivation

| **Instrument**  **Supplementary Table S1. Standardized Response Means for Patient-Reported Outcomes by Study Visit, Stratified by Disease Trajectory Class** | **Standardized Response Means [95% CI] from Baseline to Week** | | | | | | | | |
| --- | --- | --- | --- | --- | --- | --- | --- | --- | --- |
|  | **4** | **8** | **12** | **20** | **24** | **32** | **36** | **48** | **52** |
| *Class 1: Highly active, non-responder* | | | | | | | | | |
| FACIT-F | 0.21 [0.09, 0.33] | 0.23 [0.10, 0.36] | 0.28 [0.15, 0.42] | 0.45 [0.23, 0.67] | 0.30 [0.15, 0.44] | 0.50 [0.28, 0.72] | 0.41 [0.26, 0.56] | 0.64 [0.41, 0.87] | 0.49 [0.32, 0.65] |
| SF-36 PCS | 0.25 [0.08, 0.41] | 0.32 [0.11, 0.54] | 0.41 [0.19, 0.62] | 0.44 [0.16, 0.71] | 0.42 [0.20, 0.64] | 0.41 [0.12, 0.69] | 0.51 [0.28, 0.73] | 0.58 [0.29, 0.87] | 0.62 [0.39, 0.85] |
| SF-36 MCS | 0.11 [-0.06, 0.27] | 0.11 [-0.10, 0.32] | 0.12 [-0.09, 0.33] | 0.14 [-0.13, 0.40] | 0.34 [0.12, 0.55] | 0.10 [-0.18, 0.37] | 0.24 [0.03, 0.45] | 0.14 [-0.13, 0.40] | 0.35 [0.13, 0.56] |
| EQ-5D-3L | 0.05 [-0.12, 0.22] | 0.04 [-0.17, 0.26] | -0.05 [-0.26, 0.16] | -0.10 [-0.37, 0.17] | 0.22 [0.00, 0.43] | -0.07 [-0.35, 0.21] | 0.08 [-0.14, 0.29] | 0.21 [-0.06, 0.48] | 0.30 [0.08, 0.52] |
| EQ-VAS | -0.02 [-0.18, 0.15] | 0.05 [-0.16, 0.26] | 0.16 [-0.05, 0.37] | 0.11 [-0.15, 0.38] | 0.29 [0.07, 0.50] | 0.15 [-0.13, 0.43] | 0.26 [0.05, 0.48] | 0.48 [0.20, 0.76] | 0.43 [0.21, 0.65] |
| *Class 2: Highly active, responder* | | | | | | | | | |
| FACIT-F | 0.21 [0.09, 0.34] | 0.49 [0.36, 0.62] | 0.52 [0.39, 0.65] | 0.58 [0.37, 0.79] | 0.76 [0.62, 0.90] | 0.78 [0.55, 1.00] | 0.78 [0.64, 0.92] | 0.68 [0.47, 0.90] | 0.70 [0.56, 0.84] |
| SF-36 PCS | 0.22 [0.02, 0.42] | 0.52 [0.31, 0.73] | 0.69 [0.47, 0.91] | 0.87 [0.50, 1.23] | 0.80 [0.57, 1.02] | 0.98 [0.60, 1.36] | 0.86 [0.63, 1.08] | 0.99 [0.60, 1.37] | 0.79 [0.56, 1.01] |
| SF-36 MCS | 0.30 [0.10, 0.50] | 0.30 [0.10, 0.50] | 0.35 [0.15, 0.55] | 0.46 [0.13, 0.78] | 0.58 [0.37, 0.79] | 0.58 [0.24, 0.92] | 0.54 [0.33, 0.75] | 0.51 [0.18, 0.85] | 0.52 [0.31, 0.73] |
| EQ-5D-3L | 0.01 [-0.19, 0.21] | 0.19 [-0.01, 0.39] | 0.21 [0.01, 0.41] | 0.33 [0.01, 0.65] | 0.43 [0.23, 0.64] | 0.29 [-0.04, 0.61] | 0.45 [0.24, 0.65] | 0.27 [-0.05, 0.59] | 0.39 [0.19, 0.60] |
| EQ-VAS | 0.15 [-0.05, 0.35] | 0.34 [0.14, 0.55] | 0.52 [0.31, 0.72] | 0.6 [0.26, 0.93] | 0.66 [0.44, 0.87] | 0.90 [0.52, 1.27] | 0.71 [0.49, 0.93] | 0.74 [0.38, 1.09] | 0.75 [0.52, 0.97] |
| *Class 3: Moderately active, responder* | | | | | | | | | |
| FACIT-F | 0.40 [0.28, 0.53] | 0.60 [0.46, 0.74] | 0.56 [0.43, 0.70] | 0.66 [0.46, 0.86] | 0.64 [0.50, 0.78] | 0.65 [0.45, 0.85] | 0.60 [0.46, 0.74] | 0.61 [0.41, 0.81] | 0.60 [0.45, 0.74] |
| SF-36 PCS | 0.36 [0.18, 0.53] | 0.44 [0.25, 0.63] | 0.49 [0.30, 0.68] | 0.72 [0.40, 1.03] | 0.63 [0.43, 0.83] | 0.67 [0.35, 0.98] | 0.58 [0.38, 0.78] | 0.63 [0.32, 0.94] | 0.65 [0.45, 0.85] |
| SF-36 MCS | 0.26 [0.09, 0.43] | 0.47 [0.28, 0.66] | 0.49 [0.30, 0.69] | 0.58 [0.27, 0.87] | 0.49 [0.30, 0.68] | 0.63 [0.31, 0.94] | 0.65 [0.45, 0.85] | 0.57 [0.26, 0.87] | 0.62 [0.42, 0.82] |
| EQ-5D-3L | 0.38 [0.20, 0.56] | 0.39 [0.19, 0.58] | 0.53 [0.33, 0.73] | 0.56 [0.25, 0.86] | 0.55 [0.35, 0.75] | 0.69 [0.36, 1.01] | 0.59 [0.39, 0.79] | 0.73 [0.41, 1.05] | 0.60 [0.40, 0.80] |
| EQ-VAS | 0.37 [0.20, 0.55] | 0.53 [0.34, 0.73] | 0.61 [0.41, 0.80] | 0.62 [0.32, 0.92] | 0.63 [0.42, 0.82] | 0.58 [0.27, 0.89] | 0.64 [0.43, 0.83] | 0.65 [0.34, 0.96] | 0.79 [0.58, 1.00] |
| *Class 4: Moderately active, non-responder* | | | | | | | | | |
| FACIT-F | 0.33 [0.27, 0.39] | 0.4 [0.33, 0.46] | 0.38 [0.31, 0.44] | 0.39 [0.30, 0.47] | 0.37 [0.30, 0.43] | 0.40 [0.31, 0.49] | 0.39 [0.32, 0.45] | 0.42 [0.33, 0.51] | 0.46 [0.39, 0.53] |
| SF-36 PCS | 0.25 [0.17, 0.32] | 0.35 [0.26, 0.44] | 0.38 [0.29, 0.46] | 0.44 [0.30, 0.57] | 0.44 [0.35, 0.53] | 0.43 [0.29, 0.57] | 0.46 [0.37, 0.55] | 0.55 [0.40, 0.69] | 0.50 [0.41, 0.59] |
| SF-36 MCS | 0.22 [0.14, 0.30] | 0.26 [0.17, 0.34] | 0.26 [0.18, 0.35] | 0.18 [0.05, 0.31] | 0.27 [0.18, 0.36] | 0.24 [0.10, 0.37] | 0.25 [0.16, 0.34] | 0.20 [0.07, 0.34] | 0.32 [0.23, 0.41] |
| EQ-5D-3L | 0.16 [0.08, 0.23] | 0.14 [0.06, 0.23] | 0.17 [0.09, 0.26] | 0.17 [0.03, 0.30] | 0.16 [0.07, 0.25] | 0.28 [0.15, 0.42] | 0.24 [0.16, 0.33] | 0.28 [0.14, 0.42] | 0.29 [0.21, 0.38] |
| EQ-VAS | 0.21 [0.14, 0.29] | 0.33 [0.25, 0.42] | 0.35 [0.26, 0.43] | 0.28 [0.14, 0.41] | 0.37 [0.29, 0.46] | 0.45 [0.31, 0.60] | 0.39 [0.30, 0.48] | 0.58 [0.43, 0.72] | 0.50 [0.41, 0.59] |
| *All Classes: Pooled Data* | | | | | | | | | |
| FACIT-F | 0.30 [0.26, 0.35] | 0.42 [0.37, 0.47] | 0.42 [0.37, 0.47] | 0.46 [0.38, 0.53] | 0.46 [0.41, 0.51] | 0.49 [0.42, 0.56] | 0.48 [0.43, 0.54] | 0.50 [0.43, 0.58] | 0.53 [0.47, 0.58] |
| SF-36 PCS | 0.26 [0.2, 0.32] | 0.38 [0.31, 0.45] | 0.43 [0.36, 0.51] | 0.52 [0.41, 0.63] | 0.51 [0.44, 0.58] | 0.52 [0.41, 0.63] | 0.53 [0.46, 0.60] | 0.61 [0.50, 0.72] | 0.57 [0.49, 0.64] |
| SF-36 MCS | 0.22 [0.16, 0.28] | 0.28 [0.21, 0.35] | 0.29 [0.22, 0.36] | 0.26 [0.16, 0.36] | 0.34 [0.27, 0.41] | 0.3 [0.19, 0.41] | 0.34 [0.27, 0.41] | 0.28 [0.17, 0.38] | 0.39 [0.32, 0.46] |
| EQ-5D-3L | 0.16 [0.10, 0.22] | 0.17 [0.11, 0.24] | 0.20 [0.13, 0.27] | 0.19 [0.09, 0.30] | 0.25 [0.18, 0.31] | 0.27 [0.17, 0.38] | 0.29 [0.22, 0.36] | 0.33 [0.22, 0.44] | 0.35 [0.27, 0.42] |
| EQ-VAS | 0.19 [0.13, 0.25] | 0.33 [0.26, 0.40] | 0.38 [0.31, 0.45] | 0.34 [0.23, 0.44] | 0.43 [0.36, 0.50] | 0.47 [0.36, 0.58] | 0.45 [0.38, 0.52] | 0.59 [0.47, 0.70] | 0.56 [0.48, 0.63] |

| Small effect (SRM ≥0.2) | Moderate effect (SRM ≥0.5) | Large effect (SRM ≥0.8) |
| --- | --- | --- |

**Legend.** Standardized response means (SRMs) with confidence intervals (CI) are presented for the FACIT-F, SF-36 PCS, SF-36 MCS, EQ-5D-3L, and EQ-VAS at each timepoint (baseline to weeks 4 through 52), both in the pooled population and within each of the four latent trajectory classes: class 1, highly active non-responder; class 2, highly active responder; class 3, moderately active responder; and class 4, moderately active non-responder. SRMs are color coded by magnitude of effect size: ≥0.2, small;  ≥0.5, moderate; and ≥0.8, large.

Abbreviations: *EQ-5D-3L*, EuroQol 5-Dimension 3-level; *EQ-VAS*, EuroQol Visual Analogue Scale; *FACIT-F*, Functional Assessment of Chronic Illness Therapy–Fatigue; *MCS*, Mental Component Summary; *PCS*, Physical Component Summary; *PGA,* Physician’s Global Assessment; *PRO*, patient-reported outcome; *SF-36*, 36-Item Short Form Survey; *SRM,* standardized response mean.

**Supplemental Table S2. Mean Changes in Patient-Reported Outcomes Relative to Established Minimal Clinically Important Difference Thresholds**

| **Outcome** | **Responder Pathway** | | | | **Non-Responder Pathway** | | | |
| --- | --- | --- | --- | --- | --- | --- | --- | --- |
|  | **Class 2** | | **Class 3** | | **Class 1** | | **Class 4** | |
|  | **Week 24** | **Week 52** | **Week 24** | **Week 52** | **Week 24** | **Week 52** | **Week 24** | **Week 52** |
| FACIT-F | 6.00 | 7.00 | 7.00 | 7.08 | 5.00 | 5.50 | 3.00 | 4.39 |
| SF-36 PCS | 4.00 | 7.00 | 5.00 | 5.79 | 3.01 | 4.56 | 3.02 | 3.71 |
| EQ-VAS | 9.00 | 15.0 | 13.0 | 16.0 | 6.00 | 9.00 | 7.00 | 9.00 |
| EQ-5D-3L | 0.12 | 0.00 | 0.09 | 0.10 | 0.04 | 0.05 | 0.03 | 0.05 |

**Legend.** Mean changes from baseline to weeks 24 and 52 are presented for patient-reported outcome (PRO) measures across responder (classes 2 and 3) and non-responder (classes 1 and 4) disease trajectory pathways. Values are interpreted relative to established minimal clinically important difference (MCID) thresholds to assess the clinical meaningfulness of observed changes over time. Established MCID thresholds were 4 points for FACIT-F, 2.5 points for SF-36 PCS, 10 points for EQ-VAS and 0.04 for EQ-5D-3L in systemic lupus erythematosus (SLE).

Abbreviations: *EQ-5D-3L*, EuroQol 5-Dimension 3-level; *EQ-VAS*, EuroQol Visual Analogue Scale; *FACIT-F*, Functional Assessment of Chronic Illness Therapy–Fatigue; *MCID*, minimal clinically important difference*; MCS*, Mental Component Summary; *PCS*, Physical Component Summary; *SF-36*, 36-Item Short Form.

**Supplementary Table S3. Participant Follow-up and Data Availability by Study Visit**

| **Study Visit (Week)** | **Sample Size, n (%)** | | | | |
| --- | --- | --- | --- | --- | --- |
|  | **Class 1** | **Class 2** | **Class 3** | **Class 4** | **All Classes** |
| 0 | 274 | 259 | 254 | 1121 | 1908 |
| 4 | 274 (100%) | 259 (100%) | 254 (100%) | 1121 (100%) | 1908 (100%) |
| 8 | 220 (80.29%) | 259 (100%) | 236 (92.91%) | 964 (85.99%) | 1679 (88.00%) |
| 12 | 211 (77.01%) | 259 (100%) | 235 (92.52%) | 954 (85.10%) | 1659 (86.95%) |
| 20 | 88 (32.12%) | 100 (38.61%) | 116 (45.67%) | 536 (47.81%) | 840 (44.03%) |
| 24 | 198 (72.26%) | 257 (99.23%) | 230 (90.55%) | 935 (83.41%) | 1620 (84.91%) |
| 32 | 89 (32.48%) | 100 (38.61%) | 117 (46.06%) | 537 (47.90%) | 843 (44.18%) |
| 36 | 180 (65.69%) | 250 (96.53%) | 226 (88.98%) | 912 (81.36%) | 1568 (82.18%) |
| 48 | 88 (32.12%) | 101 (39.00%) | 116 (45.67%) | 540 (48.17%) | 845 (44.29%) |
| 52 | 165 (60.22%) | 242 (93.44%) | 224 (88.19%) | 876 (78.14%) | 1507 (78.98%) |

**Legend.** The number and proportion of participants with available patient-reported outcome and clinician-assessed disease activity data are reported for each study visit from baseline through week 52. Percentages for each trajectory class are calculated relative to the baseline number of participants in that class, and percentages for all classes are calculated relative to the full baseline analytic cohort. These data reflect follow-up completeness across trial visits.

**Supplementary Table S4. Baseline Participant Characteristics for Complete versus Incomplete Follow-up**

| **Variable** | **Incomplete-Follow-up**  **(N=401)** | **Complete-Follow-up**  **(52 weeks, N=1507)** | **P-value** |  |
| --- | --- | --- | --- | --- |
|  |  |  |  |  |
| BILAG | 18.00 [11.00, 22.00] | 17.00 [11.00, 21.00] | 0.03 |  |
| SLEDAI-2K | 10.00 [8.00, 12.00] | 10.00 [8.00, 12.00] | 0.02 |  |
| PGA | 1.50 [1.14, 1.95] | 1.47 [1.11, 1.83] | 0.07 |  |
| FACIT-F | 29.00 [18.00, 40.00] | 32.00 [22.00, 40.00] | 0.001 |  |
| Female | 381 (95.01%) | 1428 (94.76%) | 0.94 |  |
| Male | 20 (4.99%) | 79 (5.24%) | 0.94 |  |
| Age | 35.00 [27.00, 46.00] | 37.00 [29.00, 46.00] | 0.03 |  |
| SF-36 PCS | 36.01 [30.16, 43.65] | 40.09 [33.37, 46.33] | < 0.001 |  |
| SF-36 MCS | 41.58 [32.61, 49.72] | 40.51 [33.65, 48.69] | 0.93 |  |
| EQ-5D-3L | 0.77 [0.60, 0.82] | 0.80 [0.71, 0.83] | < 0.001 |  |
| EQ-VAS | 60.00 [50.00, 75.00] | 68.50 [50.00, 80.00] | 0.05 |  |

**Legend.** Baseline demographic and clinical variables are summarized for participants with complete and incomplete follow-up through week 52. Continuous variables are presented as median [Q1, Q3], and categorical variables are presented as n (%). Between-group differences were assessed using Wilcoxon rank-sum tests for continuous variables and chi-squared tests for categorical variables; corresponding p-values are shown to evaluate whether baseline characteristics differed by follow-up status and to assess the potential impact of missing data on study findings.

Abbreviations: *BILAG*, British Isles Lupus Assessment Group index; *CI,* confidence interval; *EQ-5D-3L*, EuroQol 5-Dimension 3-level; *EQ-VAS*, EuroQol Visual Analogue Scale; *FACIT-F*, Functional Assessment of Chronic Illness Therapy–Fatigue; *MCS*, Mental Component Summary; *PCS*, Physical Component Summary; *PGA,* Physician’s Global Assessment; *SF-36*, 36-Item Short Form Survey;  *SLEDAI-2K*, Systemic Lupus Erythematosus Disease Activity Index 2000.

**Supplementary Table S5. Responsiveness of Patient-Reported Outcomes at Weeks 24 and 52 Using Base Case, Multiple Imputation, and Classes Excluding FACIT-F from Trajectory Derivation**

| **Instrument** | **Standardized Response Means [95% CI]** | | | | | | |
| --- | --- | --- | --- | --- | --- | --- | --- |
|  | **Base Case Data (n= 1908)** | | **Multiple Imputation (n= 1908)** | | **Classes without FACIT Contribution (n =1908)** | | |
|  | **Week 24** | **Week 52** | **Week 24** | **Week 52** | **Week 24** | **Week 52** |  |
| *Class 1: Highly active, non-responder* | | | | | | | |
| FACIT-F | 0.30 [0.15, 0.44] | 0.49 [0.32, 0.65] | 0.32 [0.19, 0.45] | 0.43 [0.29, 0.57] | 0.96 [0.70, 1.22] | 0.71 [0.46, 0.96] |  |
| SF-36 PCS | 0.42 [0.20, 0.64] | 0.62 [0.39, 0.85] | 0.43 [0.15, 0.70] | 0.51 [0.28, 0.75] | 0.93 [0.52, 1.33] | 0.88 [0.48, 1.27] |  |
| SF-36 MCS | 0.34 [0.12, 0.55] | 0.35 [0.13, 0.56] | 0.35 [0.06, 0.63] | 0.35 [0.11, 0.60] | 0.68 [0.30, 1.05] | 0.60 [0.23, 0.96] |  |
| EQ-5D-3L | 0.22 [0.00, 0.43] | 0.30 [0.08, 0.52] | 0.28 [0.05, 0.52] | 0.35 [0.09, 0.61] | 0.68 [0.30, 1.04] | 0.71 [0.33, 1.09] |  |
| EQ-VAS | 0.29 [0.07, 0.50] | 0.43 [0.21, 0.65] | 0.25 [-0.04, 0.55] | 0.43 [0.15, 0.71] | 0.96 [0.70, 1.22] | 0.71 [0.46, 0.96] |  |
| *Class 2: Highly active, responder* | | | | | | | |
| FACIT-F | 0.76 [0.62, 0.90] | 0.70 [0.56, 0.84] | 0.75 [0.61, 0.89] | 0.70[0.57, 0.84] | 0.63 [0.49, 0.76] | 0.63 [0.49, 0.76] |  |
| SF-36 PCS | 0.80 [0.57, 1.02] | 0.79 [0.56, 1.01] | 0.80 [0.57, 1.02] | 0.79 [0.57, 1.01] | 0.66 [0.45, 0.86] | 0.64 [0.43, 0.84] |  |
| SF-36 MCS | 0.58 [0.37, 0.79] | 0.52 [0.31, 0.73] | 0.58 [0.37, 0.79] | 0.52 [0.31, 0.73] | 0.54 [0.33, 0.74] | 0.48 [0.28, 0.68] |  |
| EQ-5D-3L | 0.43 [0.23, 0.64] | 0.39 [0.19, 0.60] | 0.43 [0.23, 0.64] | 0.39 [0.19, 0.60] | 0.32 [0.12, 0.51] | 0.27 [0.07, 0.46] |  |
| EQ-VAS | 0.66 [0.44, 0.87] | 0.75 [0.52, 0.97] | 0.66 [0.44, 0.87] | 0.75 [0.53, 0.97] | 0.63 [0.42, 0.83] | 0.70 [0.49, 0.91] |  |
| *Class 3: Moderately active, responder* | | | | | | | |
| FACIT-F | 0.64 [0.50, 0.78] | 0.60 [0.45, 0.74] | 0.63 [0.49, 0.77] | 0.60 [0.46, 0.74] | 0.50 [0.39, 0.62] | 0.57 [0.44, 0.69] |  |
| SF-36 PCS | 0.63 [0.43, 0.83] | 0.65 [0.45, 0.85] | 0.65 [0.47, 0.84] | 0.71 [0.52, 0.90] | 0.52 [0.35, 0.68] | 0.56 [0.39, 0.72] |  |
| SF-36 MCS | 0.49 [0.30, 0.68] | 0.62 [0.42, 0.82] | 0.50 [0.30, 0.70] | 0.63 [0.44, 0.82] | 0.37 [0.22, 0.53] | 0.51 [0.35, 0.67] |  |
| EQ-5D-3L | 0.55 [0.35, 0.75] | 0.60 [0.40, 0.80] | 0.57 [0.36, 0.79] | 0.62 [0.42, 0.81] | 0.37 [0.21, 0.53] | 0.48 [0.32, 0.64] |  |
| EQ-VAS | 0.63 [0.42, 0.82] | 0.79 [0.58, 1.00] | 0.62 [0.41, 0.82] | 0.76 [0.53, 1.00] | 0.52 [0.36, 0.68] | 0.81 [0.63, 0.98] |  |
| *Class 4: Moderately active, non-responder* | | | | | | | |
| FACIT-F | 0.37 [0.30, 0.43] | 0.46 [0.39, 0.53] | 0.34 [0.27, 0.41] | 0.42 [0.35, 0.49] | 0.36 [0.29, 0.42] | 0.47 [0.40, 0.54] |  |
| SF-36 PCS | 0.44 [0.35, 0.53] | 0.50 [0.41, 0.59] | 0.40 [0.28, 0.52] | 0.46 [0.36, 0.56] | 0.45 [0.36, 0.54] | 0.54 [0.44, 0.63] |  |
| SF-36 MCS | 0.27 [0.18, 0.36] | 0.32 [0.23, 0.41] | 0.25 [0.15, 0.34] | 0.26 [0.17, 0.34] | 0.28 [0.19, 0.37] | 0.31 [0.23, 0.40] |  |
| EQ-5D-3L | 0.16 [0.07, 0.25] | 0.29 [0.21, 0.38] | 0.18 [0.10, 0.26] | 0.30 [0.21, 0.39] | 0.17 [0.09, 0.26] | 0.30 [0.21, 0.38] |  |
| EQ-VAS | 0.37 [0.29, 0.46] | 0.50 [0.41, 0.59] | 0.35 [0.23, 0.47] | 0.45 [0.35, 0.55] | 0.36 [0.27, 0.45] | 0.47 [0.38, 0.56] |  |

| Small effect (SRM ≥0.2) | Moderate effect (SRM ≥0.5) | Large effect (SRM ≥0.8) |
| --- | --- | --- |

**Legend.** Standardized response means for patient-reported outcomes in the overall cohort and stratified by disease trajectory class (class 1, highly active non-responder; class 2, highly active responder; class 3, moderately active responder; class 4, moderately active non-responder). Results are presented across six columns corresponding to raw data, multiple imputation by chained equations (MICE), and a sensitivity analysis excluding FACIT-F from disease trajectory class derivation. Each analytic approach reports SRMs at weeks 24 and 52. MICE analyses account for missing data. Effect size magnitude is indicated by color coding: 0.2 or greater, small; 0.5 or greater, moderate; and 0.8 or greater, large.

Abbreviations: *CI*, confidence interval; *EQ-5D-3L*, EuroQol 5-Dimension 3-level; *EQ-VAS*, EuroQol Visual Analogue Scale; *FACIT-F*, Functional Assessment of Chronic Illness Therapy–Fatigue; *MCS*, Mental Component Summary; *PCS*, Physical Component Summary; *PGA*, Physician’s Global Assessment; *PRO*, patient-reported outcome; *SF-36*, 36-Item Short Form Survey; *SRM*, standardized response mean.

**Supplementary Table S6. Repeated-Measures Correlations Between Patient-Reported Outcomes and Disease Activity: Sensitivity Analysis using Multiple Imputation**

| *Correlation [95% CIs* | **Clinician Reported** | | | **Patient Reported Instruments** | | | | |
| --- | --- | --- | --- | --- | --- | --- | --- | --- |
|  | BILAG | SLEDAI-2K | PGA | FACIT-F | SF-36 PCS | SF-36 MCS | EQ-5D-3L | EQ-VAS |
| ***Class 1 and 2: Highly Active*** | | | | | | | | |
|  | *Class 1: Highly active, non-responder* | | | | | | | |
| BILAG |  | 0.48 [0.45, 0.51] | 0.44 [0.41, 0.47] | -0.16 [-0.2, -0.11] | -0.17 [-0.23, -0.11] | -0.06 [-0.12, 0] | -0.14 [-0.19, -0.09] | -0.18 [-0.23, -0.12] |
| SLEDAI-2K | 0.74 [0.73, 0.76] |  | 0.43 [0.39, 0.46] | -0.09 [-0.14, -0.04] | -0.10 [-0.15, -0.05] | -0.05 [-0.11, 0.00] | -0.09 [-0.14, -0.03] | -0.14 [-0.21, -0.07] |
| PGA | 0.69 [0.67, 0.71] | 0.73 [0.71, 0.74] |  | -0.22 [-0.26, -0.17]] | -0.22 [-0.28, -0.16] | -0.12 [-0.17, -0.07] | -0.17 [-0.22, -0.12] | -0.23 [-0.3, -0.16] |
| FACIT-F | -0.26 [-0.29, -0.23] | -0.23 [-0.27, -0.19] | -0.29 [-0.33, -0.25] |  | 0.64 [0.56, 0.73] | 0.24 [0.08, 0.40] | 0.49 [0.42, 0.57] | 0.47 [0.34, 0.60] |
| SF-36 PCS | -0.13 [-0.24, -0.01] | -0.11 [-0.20, -0.02] | -0.13 [-0.22, -0.03] | 0.23 [-0.19, 0.65] |  | -0.05 [-0.15, 0.05] | 0.46 [0.39, 0.54] | 0.43 [0.31, 0.56] |
| SF-36 MCS | -0.10 [-0.21, 0.01] | -0.09 [-0.20, 0.01] | -0.10 [-0.21, 0.00] | 0.25 [0.03, 0.47] | -0.21 [-0.52, 0.11] |  | 0.30 [0.20, 0.39] | 0.19 [0.09, 0.29] |
| EQ-5D-3L | -0.14 [-0.21, -0.08] | -0.11 [-0.19, -0.04] | -0.15 [-0.23, -0.07] | 0.41 [0.26, 0.55] | 0.39 [0.24, 0.53] | 0.23 [0.1, 0.37] |  | 0.38 [0.25, 0.51] |
| EQ-VAS | -0.19 [-0.28, -0.10] | -0.20 [-0.27, -0.12] | -0.22 [-0.32, -0.12] | 0.27 [0.06, 0.47] | 0.24 [0.01, 0.46] | 0.18 [0.07, 0.29] | 0.25 [0.09, 0.41] |  |
|  | *Class 2: Highly active, responder* | | | | | | | |
| ***Class 3 and 4: Moderately Active*** | | | | | | | | |
|  | *Class 3: Moderately active, responder* | | | | | | | |
| BILAG |  | 0.66 [0.64, 0.68] | 0.60 [0.57, 0.62] | -0.23 [-0.27, -0.18] | -0.15 [-0.22, -0.08] | -0.13 [-0.18, -0.08] | -0.13 [-0.22, -0.04] | -0.11 [-0.17, -0.05] |
| SLEDAI-2K | 0.49 [0.48, 0.50] |  | 0.61 [0.58, 0.63] | -0.20 [-0.25, -0.16] | -0.11 [-0.15, -0.06] | -0.14 [-0.19, -0.09] | -0.08 [-0.14, -0.02] | -0.08 [-0.15, -0.02] |
| PGA | 0.49 [0.48, 0.50] | 0.43 [0.42, 0.45] |  | -0.25 [-0.29, -0.21] | -0.16 [-0.23, -0.09] | -0.18 [-0.22, -0.13] | -0.15 [-0.25, -0.05] | -0.12 [-0.2, -0.05] |
| FACIT-F | -0.13 [-0.15, -0.11] | -0.09 [-0.11, -0.07] | -0.14 [-0.16, -0.12] |  | 0.51 [0.40, 0.62] | 0.36 [0.20, 0.52] | 0.34 [0.24, 0.44] | 0.17 [0.05, 0.30] |
| SF-36 PCS | -0.14 [-0.16, -0.11] | -0.08 [-0.11, -0.05] | -0.13 [-0.17, -0.10] | 0.49 [0.45, 0.52] |  | -0.02 [-0.09, 0.06] | 0.41 [0.31, 0.51] | 0.16 [0.04, 0.28] |
| SF-36 MCS | -0.08 [-0.11, -0.06] | -0.07 [-0.1, -0.04] | -0.11 [-0.13, -0.09] | 0.52 [0.48, 0.56] | -0.02 [-0.07, 0.04] |  | 0.16 [0.04, 0.28] | 0.11 [0.05, 0.17] |
| EQ-5D-3L | -0.12 [-0.15, -0.09] | -0.07 [-0.09, -0.04] | -0.10 [-0.13, -0.08] | 0.48 [0.45, 0.51] | 0.46 [0.42, 0.5] | 0.36 [0.34, 0.38] |  | 0.16 [0.06, 0.27] |
| EQ-VAS | -0.13 [-0.15, -0.10] | -0.10 [-0.13, -0.07] | -0.15 [-0.18, -0.12] | 0.46 [0.42, 0.51] | 0.38 [0.34, 0.43] | 0.29 [0.25, 0.33] | 0.39 [0.35, 0.43] |  |
|  | *Class 4: Moderately active, non-responder* | | | | | | | |

| High (-0.70, -1.00) | Moderate (-0.50, -0.69) | Low (-0.30, -0.49) | Negligible (-0.29, 0.29) | Low (0.30, 0.49) | Moderate (0.50, 0.69) | High (0.70, 1.00) |
| --- | --- | --- | --- | --- | --- | --- |

**Legend**. Values represent repeated-measures correlations coefficients [95% confidence intervals] between changes in patient-reported outcomes and concurrent changes in clinician-assessed disease activity measures from baseline through week 52. Results are presented using multiple imputation by chained equations (MICE) as a sensitivity analysis and are stratified by disease trajectory class. For highly active groups (classes 1 and 2), class 1 (highly active non-responder) values are shown in the upper triangle and class 2 (highly active responder) values in the lower triangle. For moderately active groups (classes 3 and 4), class 3 (moderately active responder) values are presented in the upper triangle and class 4 (moderately active non-responder) values in the lower triangle. Negative correlation coefficients indicate that increases in disease activity are associated with worsening patient-reported outcomes.

Abbreviations: *BILAG*, British Isles Lupus Assessment Group index; *EQ-5D-3L*, EuroQol 5-Dimension 3-level; *EQ-VAS*, EuroQol Visual Analogue Scale; *FACIT-F*, Functional Assessment of Chronic Illness Therapy–Fatigue; *MCS*, Mental Component Summary; *PCS*, Physical Component Summary; *PGA*, Physician’s Global Assessment; *PRO*, patient-reported outcome; *SF-36*, 36-Item Short Form Survey; *SLEDAI-2K*, Systemic Lupus Erythematosus Disease Activity Index 2000.

**Supplementary Table S7. Repeated-Measures Correlations Between Patient-Reported Outcomes and Disease Activity: Sensitivity Analysis Excluding FACIT-F from Trajectory Class Derivation**

| *Correlation [95% CIs]* | **Clinician Reported** | | | **Patient Reported Instruments** | | | | |
| --- | --- | --- | --- | --- | --- | --- | --- | --- |
|  | BILAG | SLEDAI-2K | PGA | FACIT-F | SF-36 PCS | SF-36 MCS | EQ-5D-3L | EQ-VAS |
| ***Class 1 and 2: Highly Active*** | | | | | | | | |
|  | *Class 1: Highly active, non-responder* | | | | | | | |
| BILAG |  | 0.75 [0.73, 0.78] | 0.72 [0.69, 0.74] | -0.41 [-0.47, -0.35] | -0.51 [-0.60, -0.41] | -0.25 [-0.36, -0.12] | -0.36 [-0.47, -0.25] | -0.31 [-0.42, -0.19] |
| SLEDAI-2K | 0.72 [0.71, 0.74] |  | 0.72 [0.69, 0.75] | -0.35 [-0.41, -0.29] | -0.42 [-0.52, -0.31] | -0.27 [-0.38, -0.15] | -0.33 [-0.44, -0.22] | -0.22 [-0.34, -0.10] |
| PGA | 0.67 [0.65, 0.69] | 0.71 [0.70, 0.73] |  | -0.46 [-0.52, -0.40] | -0.49 [-0.58, -0.39] | -0.31 [-0.42, -0.19] | -0.40 [-0.50, -0.28] | -0.44[-0.53, -0.33] |
| FACIT-F | -0.34 [-0.37, -0.30] | -0.33 [-0.37, -0.30] | -0.39 [-0.42, -0.35] |  | 0.60 [0.51, 0.67] | 0.60 [0.51, 0.67] | 0.46 [0.35, 0.55] | 0.63 [0.54, 0.70] |
| SF-36 PCS | -0.39 [-0.45, -0.33] | -0.39 [-0.45, -0.33] | -0.44 [-0.50, -0.39] | 0.55 [0.50, 0.60] |  | 0.10 [-0.03, 0.22] | 0.44 [0.33, 0.54] | 0.51 [0.41, 0.60] |
| SF-36 MCS | -0.29 [-0.35, -0.22] | -0.24 [-0.30, -0.17] | -0.31 [-0.37, -0.24] | 0.50 [0.45, 0.55] | 0.10 [0.03, 0.17] |  | 0.32 [0.20, 0.43] | 0.36 [0.24, 0.46] |
| EQ-5D-3L | -0.23 [-0.29, -0.16] | -0.21 [-0.27, -0.14] | -0.27 [-0.33, -0.21] | 0.46 [0.40, 0.51] | 0.42 [0.36, 0.48] | 0.27 [0.20, 0.33] |  | 0.38 [0.27, 0.48] |
| EQ-VAS | -0.38 [-0.43, -0.31] | -0.37 [-0.43, -0.31] | -0.43 [-0.48, -0.37] | 0.51 [0.45, 0.55] | 0.48 [0.42, 0.53] | 0.37 [0.31, 0.43] | 0.37 [0.31, 0.43] |  |
|  | *Class 2: Highly active, responder* | | | | | | | |
| ***Class 3 and 4: Moderately Active*** | | | | | | | | |
|  | *Class 3: Moderately active, responder* | | | | | | | |
| BILAG |  | 0.60 [0.58, 0.62] | 0.51 [0.48, 0.53] | -0.24 [-0.27, -0.20] | -0.27 [-0.32, -0.22] | -0.20 [-0.25, -0.14] | -0.19 [-0.24, -0.14] | -0.28 [-0.33, -0.23] |
| SLEDAI-2K | 0.47 [0.46, 0.48] |  | 0.53 [0.51, 0.56] | -0.21 [-0.24, -0.17] | -0.24 [-0.30, -0.19] | -0.20 [-0.25, -0.15] | -0.13 [-0.18, -0.07] | -0.27 [-0.32, -0.22] |
| PGA | 0.39 [0.38, 0.41] | 0.41 [0.39, 0.42] |  | -0.30 [-0.33, -0.27] | -0.34 [-0.39, -0.29] | -0.28 [-0.33, -0.23] | -0.21 [-0.26, -0.16] | -0.36 [-0.41, -0.31] |
| FACIT-F | -0.18 [-0.19, -0.16] | -0.14 [-0.15, -0.12] | -0.22 [-0.24, -0.20] |  | 0.51 [0.47, 0.55] | 0.61 [0.58, 0.65] | 0.42 [0.38, 0.47] | 0.48 [0.44, 0.52] |
| SF-36 PCS | -0.20 [-0.23, -0.17] | -0.17 [-0.20, -0.14] | -0.28 [-0.31, -0.25] | 0.45 [0.43, 0.48] |  | 0.10 [0.05, 0.16] | 0.35 [0.30, 0.40] | 0.42 [0.38, 0.47] |
| SF-36 MCS | -0.16 [-0.19, -0.13] | -0.11 [-0.14, -0.08] | -0.19 [-0.22, -0.16] | 0.53 [0.51, 0.55] | 0.04 [0.01, 0.07] |  | 0.32 [0.26, 0.36] | 0.32 [0.27, 0.37] |
| EQ-5D-3L | -0.17 [-0.20, -0.14] | -0.13 [-0.16, -0.10] | -0.19 [-0.22, -0.16] | 0.42 [0.40, 0.45] | 0.34 [0.31, 0.36] | 0.33 [0.31, 0.36] |  | 0.35 [0.30, 0.40] |
| EQ-VAS | -0.20 [-0.23, -0.17] | -0.18 [-0.21, -0.15] | -0.25 [-0.28, -0.22] | 0.44 [0.42, 0.47] | 0.37 [0.34, 0.39] | 0.32 [0.30, 0.35] | 0.39 [0.36, 0.41] |  |
|  | *Class 4: Moderately active, non-responder* | | | | | | | |

| High (-0.70, -1.00) | Moderate (-0.50, -0.69) | Low (-0.30, -0.49) | Negligible (-0.29, 0.29) | Low (0.30, 0.49) | Moderate (0.50, 0.69) | High (0.70, 1.00) |
| --- | --- | --- | --- | --- | --- | --- |

**Legend**. Values represent repeated-measures correlations coefficients [95% confidence intervals] between changes in patient-reported outcomes and concurrent changes in clinician-assessed disease activity measures from baseline through week 52. Results are presented as a sensitivity analysis in which FACIT-F was excluded from disease trajectory class derivation and are stratified by disease trajectory class. For highly active groups (classes 1 and 2), class 1 (highly active non-responder) values are shown in the upper triangle and class 2 (highly active responder) values in the lower triangle. For moderately active groups (classes 3 and 4), class 3 (moderately active responder) values are presented in the upper triangle and class 4 (moderately active non-responder) values in the lower triangle. Negative correlation coefficients indicate that increases in disease activity are associated with worsening patient-reported outcomes.

Abbreviations: *BILAG*, British Isles Lupus Assessment Group index; *EQ-5D-3L*, EuroQol 5-Dimension 3-level; *EQ-VAS*, EuroQol Visual Analogue Scale; *FACIT-F*, Functional Assessment of Chronic Illness Therapy–Fatigue; *MCS*, Mental Component Summary; *PCS*, Physical Component Summary; *PGA*, Physician’s Global Assessment; *PRO*, patient-reported outcome; *SF-36*, 36-Item Short Form Survey; *SLEDAI-2K*, Systemic Lupus Erythematosus Disease Activity Index 2000.

**Supplementary Figure S1. Standardized Response Means for Patient-Reported Outcomes Over 52 Weeks: Sensitivity Analysis Excluding FACIT-F from Trajectory Class Derivation**

**Legend.** Standardized response means (SRMs) for patient-reported outcomes at study visits from week 4 through week 52, stratified by disease trajectory class (Classes 1-4; FACIT-F not included in trajectory derivation) and for the overall cohort (All Classes). SRMs are defined as the mean within-person change from baseline divided by the standard deviation of that change. Higher absolute SRM values indicate greater responsiveness. Plots display longitudinal responsiveness for the FACIT-F, SF-36 PCS, SF-36 MCS, EQ-5D-3L, and EQ-VAS. Only participants with paired baseline and post-baseline observations contributed to visit-specific estimates.

Abbreviations: *EQ-5D-3L*, EuroQol 5-Dimension 3-level; *FACIT-F,* Functional Assessment of Chronic Illness Therapy–Fatigue; *MCS*, Mental Component Summary; *PCS*, Physical Component Summary; *PGA*, Physician’s Global Assessment; *PRO*, patient-reported outcome; *SF-3*6, 36-Item Short Form Survey; *SRM*, standardized response mean; *VAS*, Visual Analogue Scale.
